# Effects of Methandienone on the ovaries of rabbits in relation to the aromatase gene (CYP19A1) and oxidative markers and the possible protective role of selenium

**DOI:** 10.12688/f1000research.174480.1

**Published:** 2026-01-24

**Authors:** Mohammed Ali Shahooth, Maysam Abdulrahman Ghazi, Sabea Khamees Abed, Ahmed Emad Abood, Mustafa A. Saud

**Affiliations:** 1 University of Anbar, Ramadi, Al Anbar Governorate, Iraq; 2 University of Fallujah, Al-Fallujah, Al Anbar Governorate, Iraq

**Keywords:** Methandienone, Selenium, CYP19A1 gene

## Abstract

**Background:**

Anabolic-androgenic steroids (AAS), such as methandienone, are synthetic testosterone derivatives that are widely used for the enhancement of physical performance and the growth of muscles. This study looked at the effect of methandienone on ovarian CYP19A1 gene expression and oxidative status in rabbits. The work also examined whether supplementation with selenium could reduce the adverse effects caused by methandienone.

**Methods:**

Twenty healthy adult female rabbits were randomly assigned to four groups and maintained under uniform management for 30 days. The groups included a control, a methandienone-treated group (T1), a selenium group (T2), and a combined methandienone plus selenium group (T3). Serum oestrogens concentrations were measured using standard biochemical techniques. CYP19A1 mRNA expression in ovarian tissue and markers were quantified by established molecular and biochemical protocols.

**Results:**

Rabbits receiving methandienone alone (T1) showed a significant decline (P≤0.05) in CYP19A1 mRNA expression and serum oestrogens when compared with the control and other treatment groups. Selenium supplementation (T2) produced marked increases in both parameters relative to G1 and G3, indicating a robust corrective effect. Oxidative stress analysis revealed elevated levels of malondialdehyde (MDA) and protein carbonyl content (PCC) in the T1 group, consistent with increased oxidative stress. Intermediate oxidative stress values were observed in the T3 group, exceeding those in the T1 group but remaining below those in the T2 group.

**Conclusion:**

Methandienone appears to impair ovarian function by suppressing CYP19A1gene expression and promoting oxidative stress, leading to reduced steroidogenic activity. Selenium administration mitigates these disturbances by enhancing antioxidant defense and partially restoring gene expression and oestrogens levels. These findings highlight the potential of selenium as a supportive intervention against methandienone -induced oxidative and hormonal disruptions in rabbits.

## Introduction

Anabolic-androgenic steroids (AAS), such as methandienone, are synthetic derivatives of testosterone which are widely used to enhance physical performance and muscle growth. Although anabolic steroids have potent anabolic properties and utilized to treat senile osteoporosis and various kinds of anemia, as well as the anabolic impact associated with masculinization ( Barbonetti et al., 2020), prolonged or excessive use is associated with a number of physiological and biochemical adverse reactions, including cardiovascular dysfunction, hepatotoxicity, endocrine imbalances and reproductive disorders ( Albano et al., 2021). The female reproductive system, especially the ovaries, is particularly sensitive to hormonal disturbances and oxidative damage as a result of exposure to exogenous steroids ( Matyja et al., 2023).

Within the ovary, aromatase, which is encoded by CYP19A1, is the key enzyme in steroidogenesis, which catalyzes the conversion of androgens to oestrogens by removing C19 from testosterone. This aromatic process is essential for follicular development, ovulation and maintenance of normal ovarian circulation ( Magnani et al., 2017). Alterations in the expression of CYP19A1 gene or in its enzyme activity may alter the physiological balance of accumulation of androgens and reduce oestrogens synthesis, potentially leading to affects follicular development and oocyte maturation and infertility ( El Fouikar et al., 2024). Elevated androgen levels are also strongly associated with follicular cystic in animals ( Kalhori et al., 2018) and polycystic ovarian syndrome (PCOS), a hormonal disorder characterized by masculinity, anovulation, menstrual irregularities and multiple ovarian cysts, which often leads to infertility and widespread metabolic disorders ( Morales-Ledesma et al., 2017).

Methandienone has been shown to induce oxidative stress by inducing over production of reactive oxygen species (ROS) and reducing the activity of antioxidant enzymes, mitochondrial dysfunctions ( Abed, 2020) and stimulating pro-inflammatory cytokines (TNF-α, IL-6) ( Petrovic et al., 2022). This oxidative imbalance is a key pathogenic pathway mediating cytotoxicity and multiple organ apoptotic effects of anabolic-androgenic steroids ( Abed, 2020). In ovarian tissue, increased levels of ROS impair mitochondrial integrity, increase lipid peroxidation and induce apoptosis of granulocytes, thereby reducing both follicular development and steroidogenic synthesis ( Dutta et al., 2024).

Selenium (Se) is a basic micronutrient and a critical component of several selenoproteins such as glutathione peroxidase (GPx) and thioredoxin reductase (ThiR) and Selenoprotein P (SelP) which play a central role in maintaining the balance of redox and preserving mitochondrial integrity in the body. Experimental data suggest that selenium supplementation reduces oxidative damage by activating the Nrf2 pathway, regulates steroidogenic enzyme expression and improves mammalian reproduction ( Rabah et al., 2023). Selenium is also essential in a number of biological processes, including electron transport, regulates immune cell proliferation, hydroxylation and oxidative metabolism of aromatic compounds in the metabolism of animals ( Stolwijk et al., 2020).

The hypothesis of the study is that methandienone is expected to affect ovarian physiology via two related mechanisms: a reduction in oestrogen synthesis, and a reinforcement of oxidative stress due to the estrogen consider as an antioxidant activity, which causes cellular and molecular damage. It is thought that supplementation with selenium compensates for these disturbances by strengthening antioxidant protection and promoting normal steroidogenic function. Exploring these interactions in the rabbit model may provide valuable insights into the contribution of anabolic and androgenic steroids to ovarian dysfunction and may inform strategies for reducing their reproductive adverse effect.

## Materials and methods

In this study, we used twenty young adult female rabbits, aged between 8 and 14 weeks, weighing roughly 850 to 1,200 grams. These rabbits were kept in the Physiology Laboratory at the Veterinary Medicine College in Fallujah University, randomly divided them into four groups, with five rabbits in each. for the T1 group gave a dose of methandienone (Black dragon pharma, Thailand) orally, which was 0.35 mg/kg body weight Selenium yeast supplied by (21st Century ^®^ (USA)) and administered to T2 group at a dose of 3 μg/kg body weight ( Abed and Al-Azawi, 2020). Methandienone and selenium were administered orally (T3) to a combined group of rabbits (0.35 mg per kg bodyweight, 3 μg per kg bodyweight). All animals received oral administration every day for 30 days. After 30 days of the experiment, blood samples were taken from each rabbit by cardiac puncture for estrogen evaluation, and at the end of the study, Animal sacrifice was carried out in accordance with approved ethical standards. and ovary were isolated for gene expression evaluation. All animal experimental procedures, including anesthesia administration and blood sampling, were performed in accordance with American Veterinary Medical Association (AVMA) guidelines ( Underwood and Raymond, 2013). The estradiol test is a one-step immunoassay used to evaluate the value estradiol in serum of mammals using a universal assay protocol called chemiflex ( Fu et al., 2020).

Malondialdehyde (MDA) assay was determined by colorimetric method ( Yalçin et al., 2010), using malondialdehyde colorimetric assay kit (Elabscience china) and assay, protein carbonyl content was determined by colorimetric method ( Reznick and Packer, 1994) using protein carbonyl colorimetric assay kit (Elabscience, china).

The total RNA was extracted with a magnatronic nanosorbent kit as specified by the manufacturer (RealBest Extraction 100, Russia). The RNA integrity and purity were verified by nanodrop spectrophotometry. We used a reverse transcriptase (M-MLV) template from the Synthol (Russia) and appropriate primers to reverse transcript the RNA to cDNA for a real-time PCR (RT). Table 1 shows the primer sequences purchased all from Bioneer (Korea). We examined the expression of these genes (the genes of interest) using GAPDH as a control gene. Start ExicyclerTM 96 real-time PCR-based thermal block device and load as follows: The PCR method was used for one hour of reverse transcriptase at 50°C, five minutes at 95°C, and 47 cycles at 96°C, 46 seconds at 62°C, all repeated 45 times. Delta-delta CT has previously been reported to be used to assess the relative amount of expression of a specific gene ( Livak and Schmittgen, 2001). Statistical analysis was performed with version 21.0 of SPSS software. Data were expressed as mean ± standard error of the mean (SE) and compared to groups using (One-way) analysis of variance (ANOVA) with a P ≥ 0.05 ( Larsen et al., 1973). All experimental procedures have been carried out in accordance with institutional guidelines on the care and use of laboratory animals.

**Table 1.** The table shows the identifies of the primers and their sequence.

Primer	Sequence	
**CYP19A1 (Aromatase enzyme)**	**F**	GGAAGAATGCATCGACTTGAGTT
	**R**	GGGCCCAAAACCAAATGGT
**GAPDH (glyceraldehyde-3-phosphate dehydrogenase)**	**F**	ATGCCCCCATGTTTGTGATG
	**R**	AGGATGCGTTGCTGACAATC

F: Forword, R: Reverse.

## Results

### 1-The effect of methandienone, selenium and both on the aromatase gene expression and serum estrogen concentration

There was a statistically significant difference ( Table 2) in the levels of mRNA CYP19A1 in ovarian tissues and serum oestrogen concentrations in rabbits treated with anabolic androgenic steroids (T1) (down regulation) compared to controls and non-treated animals (T2, T3). The selenium (T2) group showed a significant (P < 0.05) increase in gene expression and oestrogen concentrations compared to T1 and T3.

**Table 2.** The effect of methandienone, selenium and both on CYP19A1 gene expression analysis (Aromatase gene) in ovary and serum estrogen concentration of different groups (T1, T2, and T3) in female rabbits.

Groups/Parameter	Control	Methandienone (T1)	Selenium (T2)	Metha. + Se-organic (T3)	LSD
CYP19A1 gene (Fold change (2^-∆∆CT))	1.20 ± 0.03 D	3.20 ± 0.12 C	6.11 ± 0.14 A	4.54 ± 0.85 B	0.33
Estrogen (pg/ml)	37.3 ± 0.3 B	18.6 ± 0.55 D	41.8 ± 0.5 A	24.3 ± 0.54 C	0.7

-Number of rabbits in each group (5), Data reflected as mean ± Standard Error (SE).

-The different capital letters indicate significant (P ≤ 0.05) changes between variation between groups.

-control = no treatment.

-T1 = Rabbits administration of methandienone at dose 0.35 mg/kg daily for 30 days.

-T2 = Rabbits administration of selenium at dose 3 μg/kg daily for 30 days.

-T3 = Rabbits administration both of methandienone and selenium.

### 2-The effect of methandienone, selenium and both on malondialdehyde and protein carbonyl content in the ovarian tissue

A significant (P < 0.05) increases in oxidative markers (MDA and protein carbonyl content) were observed in the T1 group compared to all other groups ( Table 3). The protection group (T3) also had higher values than the control group (T2) and selenium group (T1), but lower values than T1.

**Table 3.** The effect of methandienone, selenium and both of malondialdehyde (MDA) and protein carbonyl content (PCC) levels in the ovarian tissue of different groups (T1, T2, and T3) in female rabbits.

Groups/Parameter	Control	Methandienone (T1)	Selenium (T2)	Metha. + Se-organic (T3)	LSD
MDA (micro mole/L)	2.34 ± 0.83 C	5.42 ± 0.71 A	2.21 ± 0.54 C	3.92 ± 0.65 B	0.91
PCC (nmol/mgprot)	3.5 ± 0.9 C	6.45 ± 1.2 A	4.02 ± 0.72 C	5.11 ± 1.1 B	1.09

Number of rabbits in each group (5), Data reflected as mean ± Standard Error (SE).

The different capital letters indicate significant (P ≤ 0.05) variation between groups.

-control = no treatment.

-T1 = Rabbits administration of methandienone at dose 0.35 mg/kg daily for 30 days.

-T2 = Rabbits administration of selenium at dose 3 μg/kg daily for 30 days.

-T3 = Rabbits administration both of methandienone and selenium.

## Discussion

This study showed that administration of methandienone resulted in a marked decrease in mRNA expression of CYP19A1 in ovarian tissues ( Table 2) and a concurrent decrease in serum oestrogen concentrations in treated rabbits (T1 group) compared to control groups. These findings indicate that methandienone has a suppression effect on aromatase activity, resulting in a decrease in the conversion of androgens to oestrogens. The inhibition of aromatase expression observed in this study is consistent with previous studies suggesting that AAS exposure influences the hypothalamic-pituitary-gonadal (HPG) axis and ovarian steroidogenesis through the suppression of CYP19A1 transcription ( Barros-Oliveira et al., 2021; El Fouikar et al., 2024).

Notably, supplementation with selenium (T2 group) significantly restored CYP19A1 expression and serum oestrogen concentrations compared with the methandienone-treated group. This enhancement highlights the modulatory role of selenium in maintaining the function of steroidogenic enzymes, influence on the Nrf2 pathway ( Abed, 2020) and its ability to counter the oxidative stress induced by AAS. Selenium is known to increase the activity of selenoproteins, including glutathione peroxidase and thioredoxin reductase, thereby reducing the accumulation of reactive oxygen species (ROS) and protecting the cellular macromolecules from oxidative damage ( Zhang et al., 2023). Thus, the observed upregulation of CYP19A1 following selenium supplementation may reflect the preservation of mitochondrial, endoplasmic reticulum function and transcription of antioxidant enzymes, both of which are essential for steroid hormone synthesis. These findings are consistent with Rabah et al., (2023), who demonstrated that selenium supplementation enhances ovarian function and steroidogenesis under conditions of oxidative stress.

The significant increase in oxidative markers, including malondialdehyde (MDA) and protein carbonyl (PC) levels in methandienone (T1) ( Table 3) further highlights the role of oxidative stress in ovarian dysfunction. Increased lipid peroxidation and protein oxidation, associated with decreased antioxidant protection, and when oestrogen levels fall in this group, loss of oestrogenic antioxidant protection ( Ruiz-Romero et al., 2025), reflect a disruption in the balance between reactive species generation and the body’s scavenging capacity. These oxidative insults may result in apoptosis of granulosa cells, mitochondrial dysfunction and reduced follicular viability ( Daraghmeh et al., 2025). In addition, testosterone and derivatives considered to be pro-oxidants causing adverse effects on primary biomolecules ( Andrés Juan et al., 2021). Since testosterone normally increases the metabolic rate ( Stallone and Oloyo, 2023).

Partial normalization of these oxidative markers in the group of selenium-protected (T3) supports the hypothesis that selenium provides cytoprotection by stabilizing the redox homeostasis and increasing the activity of antioxidant enzymes. Although the oxidative indices in the protection group were still higher than in the controls, their decrease compared to T1 indicates that oxidative damage caused by AAS is attenuated rather than reversed.

## Conclussion

These findings together highlight a dual pathogenic mechanism of methandienone toxicity: (1) direct endocrine disruption through aromatase inhibition and CYP19A1 downregulation; and (2) indirect oxidative damage by oxidative over production and oxidative depletion of antioxidant defences. The ability of selenium to inhibit both pathways reflects its integral role in maintaining cellular homeostasis, modulating redox signaling and stabilizing steroidogenic gene transcription.

## Ethical considerations

This study was conducted in full compliance with the ethical guidelines approved by the Scientific Committee on Research Ethics of the University of Fallujah Faculty of Veterinary Medicine (Approval No: 7; 13/10 2025) ( Shahooth et al., 2025).

## Data Availability

Underlying data Zenodo: Data are available under the terms of the Creative Commons Zero “No rights reserved” data waiver (CC0 1.0 Public domain dedication). The data raw of study of Methandienone and Selenium in rabbits. https://doi.org/10.5281/zenodo.17897289 ( Shahooth et al., 2025). **Arrive checklist** https://doi.org/10.5281/zenodo.17897289 ( Shahooth et al., 2025).
