# Supplementary material for: Effects of Methandienone on the ovaries of rabbits in relation to the aromatase gene (CYP19A1) and oxidative markers and the possible protective role of selenium
Source: F1000Res. 2026 Mar 5;15:115. Originally published 2026 Jan 24. [Version 2] doi: 10.12688/f1000research.174480.2 (PMC12982979; doi:10.12688/f1000research.174480.2)
Supplement: Peer review history

# Reviewer report: Reviewer response for version 2

**DOI:** 10.5256/f1000research.197154.r464958

**Published:** 2026-04-01

**Authors:** Luke M Pelton

**Affiliations:** 1 Springfield College, Springfield, Massachusetts, USA

I thank the authors for their diligence in responding to my previous feedback. While I feel the quality of the manuscript as a whole has dramatically improved, there are a few minor points that I would like to see addressed; however, they are relatively simple fixes and I do not feel that they prohibit the manuscript from being acceptable. 

**Intro**: For Sentence 3, I apologize; I see now that my previous feedback was poorly stated on my part. The sentence should read “ *AAS have potent anabolic properties and are often utilized to treat geriatric osteoporosis and various kinds of anemia. However, in addition to their masculinizing effects (Barbonetti et al., 2020), prolonged or excessive use is associated with a number of adverse psychological and biochemical reactions, including cardiovascular dysfunction, hepatotoxicity, endocrine imbalances and reproductive disorders (Albano et al., 2021)*.”

**Methods**: Knowing that the rabbits were of identical breed, we should definitely have that exact breed named clearly; this is important for two reasons. First, any future researchers looking to replicate this study will need to use identical subjects. Second, it allows you to make a stronger argument for why a posttest-only model was chosen (i.e., clarify the logic of the homogeneous rabbit breed circumventing the need for a pretest, as all could be assumed to be similar at baseline, etc.). 

 In terms of the methandienone sourcing: I think it is prudent to clarify the specific source through which the drug was purchased by name. Again, this helps bolster the internal validity of your investigation, and would assist future researchers in replicating your methodology. Additionally, include that the compound was analyzed for purity in the Department of Analytics at the Veterinary Directorate, and that all experimental groups received the same verified preparation under controlled conditions. This should be a simple fix. 

**Results**: I am glad to see that the analysis yielded consistent findings, and I appreciate the authors’ reformatting and presentation of the findings. However: for p values reading “0.00,” we should change these to read “< 0.001” as the probability can never truly be 0. Again, this should be a very simple change. 

**Discussion**: I would actually suggest beginning the discussion by 1) restating the purpose of the investigation along with the hypothesis. This reminds the reader of the background as they then read the main findings. 

**Conclusion**: Kindly fix the spelling of the word “Conclusion”

Is the work clearly and accurately presented and does it cite the current literature?

Yes

If applicable, is the statistical analysis and its interpretation appropriate?

Partly

Are all the source data underlying the results available to ensure full reproducibility?

Yes

Is the study design appropriate and is the work technically sound?

No

Are the conclusions drawn adequately supported by the results?

Partly

Are sufficient details of methods and analysis provided to allow replication by others?

Partly

Reviewer Expertise:

Exercise physiology, reproductive endocrinology, anabolic-androgenic steroids

I confirm that I have read this submission and believe that I have an appropriate level of expertise to confirm that it is of an acceptable scientific standard, however I have significant reservations, as outlined above.

# Reviewer report: Reviewer response for version 2

**DOI:** 10.5256/f1000research.197154.r465201

**Published:** 2026-03-12

**Authors:** Mohammed Asker

**Affiliations:** 1 Mustansiriyah University, Baghdad, Iraq

The manuscript entitled “Effects of Methandienone on the ovaries of rabbits in relation to the aromatase gene (CYP19A1), oxidative markers and the possible protective role of selenium” presents an interesting and scientifically relevant investigation.

 Overall, the study is clearly written and well organized. The authors have presented the objectives, methodology, results, and discussion in a logical and coherent manner. The experimental design appears appropriate for addressing the research question, and the methods are described with sufficient clarity to allow understanding of the procedures used.

 The manuscript is written in a clear and precise scientific style, and the literature cited is relevant and supports the background of the study. In addition, the results are presented clearly and the conclusions are generally supported by the data provided.

 The work contributes useful information regarding the effects of Methandienone on ovarian function and oxidative stress markers, as well as the potential protective role of selenium. Overall, the manuscript represents a valuable contribution to the field.

Is the work clearly and accurately presented and does it cite the current literature?

Yes

If applicable, is the statistical analysis and its interpretation appropriate?

Yes

Are all the source data underlying the results available to ensure full reproducibility?

Yes

Is the study design appropriate and is the work technically sound?

Yes

Are the conclusions drawn adequately supported by the results?

Yes

Are sufficient details of methods and analysis provided to allow replication by others?

Yes

Reviewer Expertise:

NA

I confirm that I have read this submission and believe that I have an appropriate level of expertise to confirm that it is of an acceptable scientific standard.

# Reviewer report: Reviewer response for version 1

**DOI:** 10.5256/f1000research.192385.r453351

**Published:** 2026-02-16

**Authors:** Luke M Pelton

**Affiliations:** 1 Springfield College, Springfield, Massachusetts, USA

**I had the privilege of being asked to provide an open review of the recently published “Effects of Methandienone on the ovaries of rabbits in relation to the aromatase gene (CYP19A1) and oxidative markers and the possible protective role of selenium.” While I am greatly intrigued by the premise and purpose, I believe there are several crucial points that must be addressed before it is truly ready to add its findings to this area of research.**

**In the Introduction, there is a noticeable jump from the background information of the Intro to the hypothesis. WHY is this investigation being conducted? Is there some crucial gap that exists in this previous area of research? Has anything similar been conducted previously? It seems as though your research group has actually conducted similar investigations in the brain (Abed et al. 2020). Why not present that as a recent relevant finding, and use that to pose the following: Although previous research has been conducted in brain tissues (Abed et al. 2020), there have been no such investigations in the subspinal reproductive centers. Therefore, the purpose of this study is to…”**

**In the Methods: there are several elements which gravely threaten the internal validity of this investigation. Firstly, it should be indicated whether all rabbits were the same breed/genetic strain. I am assuming this is the case, but requires clarification as any preexisting between-group differences at baseline must be accounted for. To that point: I am not seeing any indication that baseline data was gathered before the supplementation protocol. If this data was not gathered, it is a fatal threat to internal validity, as there is no accounting for whether the between-groups differences were actually a result of the protocol, or due to the aforementioned preexisting group differences.**

**Finally, the use of Black Dragon Pharma: based on the name and a Google search, this seems like a UG lab (i.e. a black market trader of non-regulated AAS and PEDs) and not a reputable pharmaceutical provider. Was there any 3rd party testing conducted on this to even determine if the dosing of the compound was accurate? This is also a potentially fatal threat to the internal validity of this investigation, as it severely limits whether we can claim that the changes in groups are due to the protocol.**

**Additionally, for the benefit of the authors, I have provided minor feedback which pertains mostly to writing style, organization, etc., and should be relatively simple for the authors to address.**

**Intro: **

1. **I would like to see a supporting citation for sentence 1**
2. **After introducing the acronymic abbreviation AAS< use that for all instances going forward (i.e. sentence 2)**
3. **“Senile osteoporosis”: is this meant to be “geriatric”? Senile implies psychological/cognitive status**
4. **Sentence 3: this structure needs revision; the second phrase could better read “...as well as their masculinizing effects…” “Anabolic properties” and “anabolic impacts" are redundant and repetitive. **
5. **Should be written “...number of adverse psychological and biochemical reactions…”**
6. **Second paragraph: clarify what C19 is or the read (and then use C19 throughout)**
7. **“Elevated androgen levels are also strongly associated with follicular cystic…” What is meant by “follicular cystic”? Should this be “follicular cysts”?**
8. **Third paragraph: should start by saying “The AAS methandienone…”**
9. **Fourth paragraph: to facilitate a better transition to this point from the previous topic, I would revise this opening point sentence: “A compound which plays a central role in maintaining the balance of redox and preserving mitochondrial integrity in the body is selenium (Se), a basic micronutrient…”**

**Results:**

1. **LSD: If this is Fisher’s LSD as used as a post hoc analysis following the ANOVA, I would urge caution in its use and extrapolation. Fisher’s LSD is often likely to return a false positive via hyperinflation of alpha in its multiple comparisons. I would suggest using Tukey’s post hoc, as it is a more conservative measure (particularly given the lack of robust supporting research provided in the introduction as to provide rationale for the expected direction of change/difference; this, compounded with the previously mentioned concerns regarding the AAS dosing, present an additional threat to internal validity). I ran the analysis on my own with the raw data set you kindly provided, and found that your findings would be maintained with the use of Tukey’s post hoc. **
2. **When stating statistically significant findings: there must be some indication of the exact p value. Saying “p < 0.05” is not enough, firstly as the threshold is “p ≤ 0.05,” and secondly as it is not indicated on the table anywhere. To that point, the use of capital letters to indicate significant differences is unwieldy and confusing. Please consider using this entire point of feedback to present the group differences in a way that both clarifies your presentation of the data and makes it more easily understood by the reader. **

**Discussion**

1. **Throughout the discussion: be careful when using words like “indicates,” as they carry with them a weight of finality. Simply including the phrase “may indicate” presents your findings in a more conservative manner appropriate for conveying the findings of a single investigation.  **
2. **Paragraph 2: this is the first introduction of the Nrf2 pathway. I recommend clarifying what that is for the reader, and how it relates to the current investigation. **
3. **Paragraph 3: the final sentence of this paragraph is an incomplete thought. **

Is the work clearly and accurately presented and does it cite the current literature?

Yes

If applicable, is the statistical analysis and its interpretation appropriate?

Partly

Are all the source data underlying the results available to ensure full reproducibility?

Yes

Is the study design appropriate and is the work technically sound?

No

Are the conclusions drawn adequately supported by the results?

Partly

Are sufficient details of methods and analysis provided to allow replication by others?

Partly

Reviewer Expertise:

Exercise physiology, reproductive endocrinology, anabolic-androgenic steroids

I confirm that I have read this submission and believe that I have an appropriate level of expertise to state that I do not consider it to be of an acceptable scientific standard, for reasons outlined above.

# Response

**Published:** 2026-02-25

**Authors:** Sabea Abed

**Affiliations:** veterinary medicine, University of Fallujah, Al-Fallujah, Al Anbar Governorate, Iraq

Dear  Reviewers,

 We sincerely thank the reviewers for their valuable time, effort, and insightful comments on our manuscript titled: *“Effects of Methandienone on the ovaries of rabbits in relation to the aromatase gene (CYP19A1) and oxidative markers and the possible protective role of selenium.”*

 In response, we have carefully revised the manuscript and addressed all points raised:

**Paragraph 1**: In response, we revised the Introduction to incorporate our previous findings in brain tissues (Abed et al., 2020) and clarified that no similar investigations have been conducted in the subspinal reproductive centers. This revision clearly defines the research gap and the objective of the present study.

**Paragraph 2:** All rabbits used in this study were of the same breed and genetic background and were obtained from the Cancer Research Center, Baghdad. The animals were age- and weight-matched prior to the start of the experiment. In addition, rabbits were randomly assigned to the experimental groups to minimize potential preexisting differences between groups. The study was designed as a randomized controlled post-test experimental model; therefore, baseline measurements were not collected prior to the supplementation protocol. The inclusion of a control group together with random allocation ensures group comparability at the beginning of the experiment and allows the observed differences to be attributed to the intervention. These methodological clarifications have now been added to the Methods section

**Paragraph 3:**The methandienone used in this study was obtained from a commercial supplement provider (Gym Center Supplements) as part of the doctoral research project. To ensure accuracy and consistency, the compound was analyzed for purity in the Department of Analytics at the Veterinary Directorate. All experimental groups received the same verified preparation under controlled conditions.

**Introduction**

1. Added a supporting citation for the first sentence.
2. Introduced the acronym “AAS” and used it consistently throughout the text.
3. Replaced “senile osteoporosis” with “geriatric osteoporosis” for accuracy.
4. Revised sentence 3 to read “…as well as their masculinizing effects…” and removed redundant references to “anabolic properties.”
5. Corrected phrasing to “…number of adverse psychological and biochemical reactions…”
6. Clarified the meaning of C19 and used it consistently throughout the paragraph.
7. Replaced “follicular cystic” with “follicular cysts” for precision.
8. Revised the third paragraph to begin with “The AAS methandienone…”
9. Improved the opening sentence of the fourth paragraph to enhance transition, now reading: “A compound which plays a central role in maintaining the balance of redox and preserving mitochondrial integrity in the body is selenium (Se), a basic micronutrient…”

**Results:**

 1- Following the reviewer’s suggestion, we have replaced Fisher’s LSD post hoc analysis with Tukey’s post hoc test to provide a more conservative assessment of group differences and reduce the risk of false positives. Re-analysis confirmed that the original findings are maintained with Tukey’s test. This change has been updated in both the Results section and corresponding tables.

 2- We have revised the presentation of statistically significant findings to include exact p-values for all comparisons (p ≤ 0.05), replacing the use of capital letters in the tables. Group differences are now presented in a clearer and more reader-friendly format, enhancing transparency and interpretability.

**Discussion**

 1- We have revised the language throughout the Discussion to use more conservative phrasing, replacing terms such as “indicates” with “may indicate” to appropriately reflect the findings of a single investigation.

 2- The Nrf2 pathway is now clearly introduced and explained in paragraph 2, including its relevance to oxidative stress and the current study’s objectives.

 3- The final sentence of paragraph 3 has been corrected to complete the thought and improve clarity.

**Finally**, we sincerely thank the reviewer for their time, effort, and valuable feedback on our manuscript. Your thorough evaluation and constructive suggestions have significantly enhanced the clarity, rigor, and overall quality of our work. We have learned a great deal from your comments, which have not only improved this study but also enriched our expertise in best practices for scientific writing and research presentation.

 Thank you for your consideration.                                                   Sincerely

 Sabea Khamees Abed

# Reviewer report: Reviewer response for version 1

**DOI:** 10.5256/f1000research.192385.r453353

**Published:** 2026-02-02

**Authors:** Aamir Maqtoof Abed Al-Ghareebawi

**Affiliations:** 1 University of Al-Shatrah, Thi-Qar, Iraq

Thank you for the opportunity to review this submission. A paper submitted to the Journal is reviewed to determine whether the paper is suitable for indexing. In the review process, the originality, technical quality, and impact of the research work are critically evaluated. According to above, the article is suitable to indexing.

Is the work clearly and accurately presented and does it cite the current literature?

Yes

If applicable, is the statistical analysis and its interpretation appropriate?

Yes

Are all the source data underlying the results available to ensure full reproducibility?

Yes

Is the study design appropriate and is the work technically sound?

Yes

Are the conclusions drawn adequately supported by the results?

Yes

Are sufficient details of methods and analysis provided to allow replication by others?

Yes

Reviewer Expertise:

Animal Physiology

I confirm that I have read this submission and believe that I have an appropriate level of expertise to confirm that it is of an acceptable scientific standard.
